# A new genus of the *Hypocera* group (Diptera, Phoridae), with descriptions of two new species from China

**DOI:** 10.3897/zookeys.932.38970

**Published:** 2020-05-12

**Authors:** Guang-Chun Liu

**Affiliations:** 1 Liaoning Key Laboratory of Urban Integrated Pest Management and Ecological Security, College of Life Science and Bioengineering, Shenyang University, Shenyang 110044, China Shenyang University Shenyang China

**Keywords:** Diptera, Phoridae, *
Sinogodavaria
*, *S. multiformis*, *S. tenebrosa*, keys

## Abstract

A new genus, *Sinogodavaria***gen. nov.**, with two new species, *S. multiformis***sp. nov.** and *S. tenebrosa***sp. nov.**, is described from China. It belongs phylogenetically to the *Hypocera* group of genera. The species *Latiborophaga bathmis* Liu is transferred to the new genus. A key to species of the new genus is presented.

## Introduction

In the course of an ongoing study of Chinese scuttle flies (Diptera, Phoridae), a series of specimens show an interesting set of characters. The male is similar to *Godavaria* Brown, 1992 and *Chaetogodavaria* Liu, 1996. The wing of the female has a thickened costa like that in the genus *Latiborophaga* Brown, 1992. However, they could not be assigned to any known genus based on a combination of characters. Therefore, a new genus with two new species is proposed. It seems to belong to the *Hypocera* group based on the spinuli of the hypandrium and on the reduced lumen of the hind coxa (Brown et al. 2015). The species *Latiborophaga bathmis* Liu is transferred to the new genus. Keys to genera based on both males and females (Disney 1994) are modified, and a key to species of the new genus is presented.

## Materials and methods

Specimens were stored in 80% ethanol. The head, legs, and one wing were detached and slide mounted according to the method of Disney (1994). For study of the male and female terminalia, the terminal abdominal segments were detached from the body and placed in a 10% solution of KOH at 50 °C for 8 hours, then dropped into an 8% solution of acetic acid for 30 minutes, and transferred to distilled water for dissection. Observations were carried out under both binocular stereoscopic and compound light microscopes. Line drawings were made using a Leica M205C with a drawing tube. Photographs were taken using Leica M205A and Leica DM5500B microscopes, with the help of a CCD 450 multi-focus imaging system. The terminology follows McAlpine (1981). The type specimens are deposited in the Natural History Museum of Shenyang University (**NMSU**), Shenyang, China.

## Taxonomy

### Genus Sinogodavaria gen. nov.

Taxon classificationAnimaliaDipteraPhoridae

95E5D21B-2A5D-5474-861C-5DFFBEAD1E4A

http://zoobank.org/723D133A-6B57-4B9A-862B-23498C5C4742

#### Type species.

*Sinogodavaria multiformis* sp. nov.

#### Diagnosis.

Supra-antennal setae absent; flagellomere 1 not elongate; costa of female thickened; wing vein Rs with several fine setulae along upper side; vein Rs deflected slightly at the junction with vein M_1_; vein R_2+3_ absent; tip of vein R_4+5_ not enlarged; hind tibia with an antero-apical seta but without an antero-basal seta; male hypandrium without dense microsetae; aedeagus with a long, curled sclerotized process.

#### Description.

***Head*.** Frons generally broader than long. Median furrow present, vestigial. Supra-antennal setae absent. First and second rows of setae convex. Flagellomere 1 globose; arista sub-apical. Palpus oval, with apical setae and ventral setulae.

***Thorax*.** Propleuron of thorax with three ventral setae, two posterior setae, and scattered setulae. Anepisternum with fine setulae on upper part. Notopleura with four setae, the second being smaller than the others. Scutellum with an anterior pair of small setulae and a posterior pair of setae.

***Legs*.** Fore tibia with a dorsal seta near basal two fifths and several small setulae below it. Mid tibia with the normal basal pair of setae and an antero-apical seta. Hind tibia with two dorsal longitudinal setal palisades, one antero-apical seta, one robust ventral spur and a series of weak posterior and postero-dorsal spurs.

***Wing*.** Costa usually extending to half of wing length. Wing vein Rs with several fine setulae along upper side. Female costa thickened around junction with vein R_1_ or at the first section of costa. Vein Sc reaching vein R_1_. Axillary ridge with 4–6 long, black, feathered setae. Haltere yellowish brown, knob black.

***Abdomen*.** Female without tergite VII. Left side of epandrium slender, rounded apically; right side of epandrium large, triangular. Hypandrium with ventrally directed outer lobe covered with rounded spinuli. Aedeagus dark brown, supported by aedeagal apodeme; its left plate with a long, curled, sclerotized process. Anal tube short.

#### Etymology.

The genus name is derived from *Sino* and *Godavaria* and refers to the locality and to the relationship with the genus *Godavaria* Brown.

#### Distribution.

China (Liaoning, Hebei, Shaanxi, Sichuan).

#### Recognition.

In Disney’s (1994) male and female keys to genera, the new genus runs to couplets 9 (males) and 119 (females), respectively, both *Borophaga* Enderlein, 1924. There is no further division of the genus *Borophaga* in these keys. In the modified key to the *Borophaga* group (Bänziger and Disney 2006), the new genus runs to couplet 3 as *Peromitra* Enderlein, 1924 and *Latiborophaga* Brown, 1992. *Sinogodavaria* gen. nov. is distinguished from *Peromitra* by a subcircular and non-erect anterior ocellus and thickened costa of the female wing. It differs from *Latiborophaga* by the more-or-less straight vein Rs and the absence of an antero-basal seta on the hind tibia. It differs from the genus *Chaetogodavaria* Liu, 1996 by the absence of a long seta on the anepisternum (Liu 1996). In consideration of new data, the following modifications of the male and female keys to genera by Disney (1994) are proposed.

##### Partial modification of the key to genera (males) by Disney (1994)

**Table:** 

9	Posterior ocelli close to eye margin and ocellar triangle strongly demarcated at front by a sinuous furrow	*** Stichillus ***
–	Posterior ocelli well removed from eye margin and ocellar triangle not demarcated in this way at front	**9a**
9a	Tip of wing vein Rs thickened	*** Borophaga ***
–	Tip of wing vein Rs not thickened	**9b**
9b	Hind tibia with antero-apical seta, but without antero-basal seta	**9c**
–	Hind tibia with both antero-apical and antero-basal setae	**9e**
9c	Anepisternum with a strong seta and short setulae	*** Chaetogodavaria ***
–	Anepisternum only with short setulae	**9d**
9d	Wing vein R_1+2_ present and strongly developed	*** Godavaria ***
–	Wing vein R_1+2_ absent; aedeagus with a long, curled, sclerotized process	***Sinogodavaria* gen. nov.**
9e	Wing vein Rs deflected at mid length; anterior ocellus not elevated; hypandrium with dense microsetae	*** Latiborophaga ***
–	Wing vein Rs more or less straight; anterior ocellus elevated; hypandrium without dense microsetae	*** Peromitra ***


##### Partial modification of the key to genera (females) by Disney (1994)

**Table:** 

114	Anepisternum bare; hind tibia without longitudinal setal palisades	**115**
–	Anepisternum with setulae; hind tibia with two dorsal longitudinal setal palisades	**114a**
114a	Wing vein R_2+3_ very weak; tip of vein R_1_ thickened	***Borophaga*** (part)
–	Wing vein R_2+3_ strongly developed; tip of vein R_1_ not thickened	*** Godavaria ***
119	Posterior ocelli clearly closer to eyes than to anterior ocellus, and ocellar region usually clearly demarcated in front by a sinuous furrow	*** Stichillus ***
–	Posterior ocelli clearly closer to anterior ocellus than to eyes, and ocellar region not clearly demarcated in this way in front	**119a**
119a	Tip of vein R_1_ thickened	***Borophaga*** (part)
–	Tip of vein R_1_ not thickened	**119b**
119b	Anterior ocellus elevated, broader than high; costa not thickened	*** Peromitra ***
–	Anterior ocellus not elevated, subcircular; costa thickened	**119c**
119c	Hind tibia with antero-basal seta; vein Rs deflected at mid length	*** Latiborophaga ***
–	Hind tibia without antero-basal seta; vein Rs deflected at the junction with vein M_1_	***Sinogodavaria* gen. nov.**

##### Key to the species of *Sinogodavaria* gen. nov.

**Table:** 

1	Female	**2**
–	Male	**4**
2	Abdominal tergites light brown with some dark area; tergite III wide-mouth-urn shaped (Fig. 7)	***S. multiformis* sp. nov.**
–	Abdominal tergites uniformly dark brown; tergite III trapezoid (Fig. 20)	**3**
3	Tergite VI triangular (Fig. 28)	***S. bathmis* (Liu)**
–	Tergite VI trapezoid (Fig. 20)	***S. tenebrosa* sp. nov.**
4	Tergites with light rear band; hypandrium with a short pointed fronto-ventral process (Figs 11, 12, 13)	***S. multiformis* sp. nov.**
–	Tergites without light rear band; hypandrium with a long hockey-stick-shaped fronto-ventral process (Figs 24–26)	***S. tenebrosa* sp. nov.**

### Sinogodavaria multiformis sp. nov.

Taxon classificationAnimaliaDipteraPhoridae

5358501D-5B55-5DD5-8C91-B9C533D513F2

http://zoobank.org/ http:/FD6434B6-C277-43C4-A0F7-E4F7C53EBFC6

Figures 1–7 , 8–13

#### Specimens examined.

***Holotype***: China • ♀; Liaoning, Mt. Qianshan; 41°05'11.63″N, 123°4'49.29″E; alt. 615 m; 16 Aug. 2018; Jiao Zhang leg.; pitfall trap; Paratypes: China • 1 ♀, 1 ♂; same data as for holotype • 1 ♀; Hebei, Zhulu, Mt. Xiaowutai; 39°46'23.21″N 115°29'49.59″E; alt. 1100 m; 27 Jul. 2009; Lixin Su leg.; sweeping net • 1 ♀; Dalian, Lvshun, Mt. Laotie; 38°44'38.37″N, 121°10'34.52″E; alt. 219 m; 12 Sep. 2010; Jianfeng Wang leg.; sweeping net • 3 ♂; Liaoning, Dalian; 38°52'44.67″N, 121°41'59.57″E; alt.110 m; 30 Jul. 2005; Li Jiang, light trap • 6 ♂; Liaoning, Dalian; 38°52'44.67″N, 121°41'59.57″E; alt.110 m; 20 Jul. 2005, Hong Fang, light trap • 1 ♂; Shaanxi, Zuoshui, Mt. Shaohua; 34°21'39.27″N, 109°12'43.67″E; alt. 676 m; 19 Jul. 2013; Yunlong Cai leg.; sweeping net.

#### Diagnosis.

Female abdominal tergites yellow with some brownish patches, venter whitish yellow; tergite III wide-mouth-urn shaped, tergite VI long and triangular. Male abdominal tergites brown, tergites II–IV divided by yellow median band; hypandrium with only a short fronto-ventral process.

**Figures 1–7.**
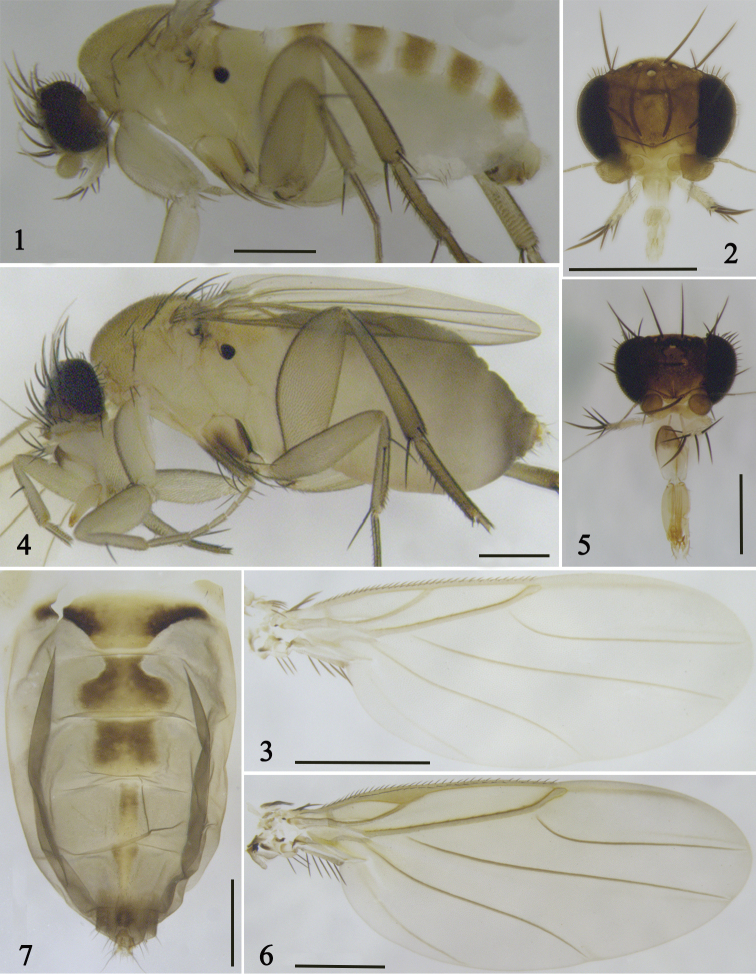
*S. multiformis* sp. nov. **1–3** male **1** lateral view of body **2** head **3** wing **4–7** female **4** lateral view of body **5** head **6** wing **7** abdominal tergites. Scale bars: 0.5 mm.

#### Description.

**Female** (Fig. 4). ***Frons*** (Fig. 5) yellowish brown, a little broader than long, with 140–150 setulae, dense microsetae. Median furrow very short, vestigial. Lower interfrontal setae near to frontal edge, clearly close to each other and ca. 1/2 × as far apart as either is from a lower fronto-orbital seta, which is much higher on frons. Upper interfrontal setae as far apart as either is from an upper fronto-orbital seta, which is slightly higher on frons. Cheek with a single seta and jowl with two long setae and some fine setulae. Flagellomere 1 globose, brown, with ca. 12 subcuticular pit sensilla; arista sub-apical, with fine microsetae; scape with dense microsetae. Palpus whitish yellow, with seven apical setae and a dozen ventral setulae. Proboscis elongate. Labrum yellow, rectangular, a little wider than diameter of flagellomere 1. Labella straw yellow, each with four setulae on upper face and a submarginal row of approximately ten small setulae below.

***Thorax*** mainly yellow-brown, paler on sides. Anepisternum with fine setulae on upper part. Notopleura with four setae, the second being smaller than the rest. Scutellum with anterior pair of small setulae and posterior pair of setae.

***Legs*** yellow. Fore tibia with a near-dorsal seta at basal 2/5 and four or five differentiated small setulae below it. Fore tarsus with a postero-dorsal longitudinal setal palisade on tarsomeres 1–3 and sometimes with a vestigial palisade in basal third of tarsomere 4. Mid tibia with the normal basal pair of setae and an antero-apical seta. Hind tibia with two dorsal longitudinal setal palisades, of which postero-dorsal one extending to 4/5 of the tibia, and with a single antero-apical seta, a robust ventral spur, and a series of weak posterior and postero-dorsal spurs.

***Wings*** (Fig. 6) 2.54 mm long. Costal index 0.57–0.58. Costal ratio 1:2.24. Costal cilia 40–43 µm long. Vein Rs with 15 or 16 pale minute setulae along upper side. Costa thickened around origin of vein R_1_. Vein Sc reaching vein R_1._ Axillary ridge with five long, black, feathered setae. Wing veins yellowish brown and membrane tinged yellowish grey. Haltere yellowish brown, knob black.

***Abdomen*** mainly yellow. Tergites (Fig. 7) yellowish brown, with different shapes. Tergite II trapezoid, with brown sides; tergite III flat, wide-mouth-urn shaped; tergite IV rectangular, tergite V thin and long, rectangular; tergite VI long and triangular. Tergites I–VI with very sparse short setulae and only a little longer at rear. The front edge of tergite II broadest, narrowing gradually posteriorly. Venter whitish yellow. Cerci yellowish brown, ca. 3 × as long as broad. The longest (apical) setulae at least 2 × as long as cercus.

**Male** (Fig. 1). ***Frons*** (Fig. 2) dark brown, a little broader than long, with 140–150 setulae and dense microsetae. Median furrow shorter than that of female. Lower interfrontal setae near to front edge, clearly close to each other and ca. 2/3 × as far apart as either is from a lower fronto-orbital seta, which is much higher on frons. Upper interfrontal setae as far apart as either is from an upper fronto-orbital seta, which is slightly higher on frons. Cheek with a single seta and jowl with two long setae and some fine setulae. Flagellomere 1 globose, dark brown, with ca. 12 subcuticular pit sensilla; arista sub-apical, with minute setulae; scape with dense microsetae. Palpus yellow, with six apical setae and a dozen ventral setulae. Proboscis shorter than in female. Labrum pale yellow and a very narrow triangle. Labella whitish yellow, each with four setulae on upper face and a submarginal row of ca. 12 small setulae below.

***Thorax*** mainly dark brown, lighter brown on sides. Anepisternum with fine setulae on upper part. Notopleura with four setae, the second being smaller than the rest. Scutellum with an anterior pair of small setulae and a posterior pair of setae.

***Legs*** yellow. Fore tibia with a near-dorsal seta at basal two fifths and eight or nine small setulae below it. Fore tarsus with a postero-dorsal longitudinal setal palisade on tarsomeres 1–3 only, plus a vestigial palisade in basal third of tarsomere 4. Mid tibia with the normal basal pair of setae and an antero-apical seta. Hind tibia with two dorsal longitudinal setal palisades, and with one antero-apical seta, one robust ventral spur, and a series of weak posterior and postero-dorsal spurs.

***Wings*** (Fig. 3) 1.70 mm long. Costal index 0.53–0.55. Costal ratio 1:1.1. Costal cilia 25–27 µm long. Vein Rs with 10–14 pale and minute setulae along upper side. Costa not thickened. Vein Sc reaching vein R_1._ Axillary ridge with five long, black, feathered setae. Wing veins yellowish brown and membrane tinged yellowish grey. Haltere yellowish brown, knob black.

***Abdominal tergites*** basically yellowish brown, front and rear margin, and middle part of each tergite yellow. Venter whitish yellow. Tergites I–VI with very sparse short setulae and only a few more setulae on tergite VI. All tergites rectangular or trapezoid.

**Figures 8–13.**
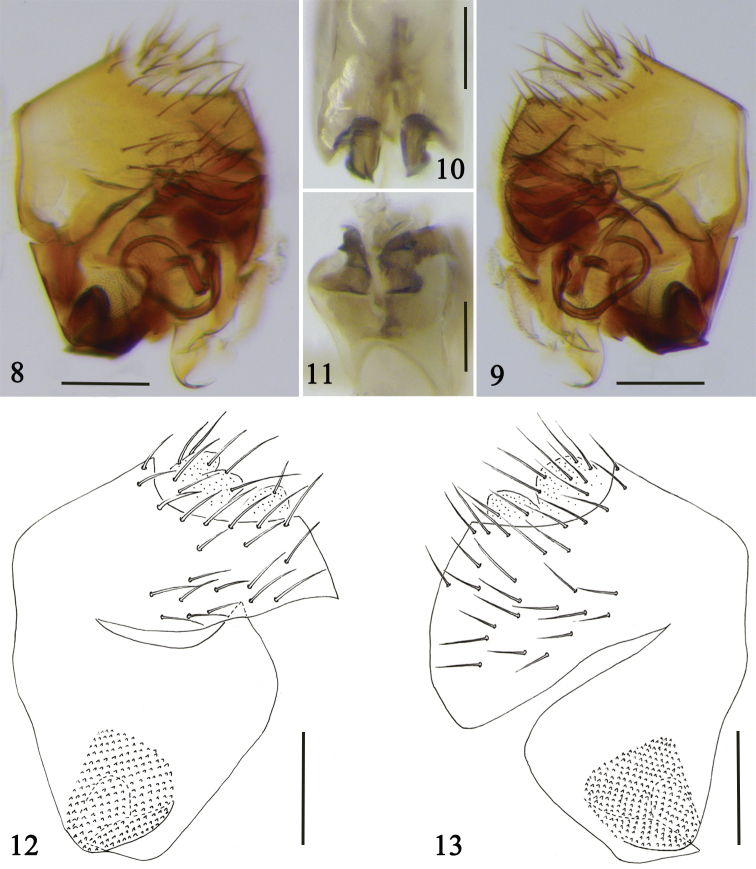
Male terminalia of *S. multiformis* sp. nov. **8, 12** left view **9, 13** right view **10** front view **11** ventral view. Scale bars: 0.1 mm.

***Male terminalia*** (Figs 8–13). Left side of epandrium slender and rounded apically, with 25–30 setulae and dense microsetae; right side of epandrium large, triangular, with some setulae. Left side of hypandrium short and wide, with a pointed upper corner. Right side of hypandrium short and rounded. A short, pointed process present at fronto-venter of each side of hypandrium. Aedeagus dark brown, supported by aedeagal apodeme and with a long, curled process. Cerci yellow, short, with long setulae.

#### Etymology.

The species name refers to the polymorphic abdominal tergites of the female.

#### Distribution.

China (Liaoning, Hebei, Shaanxi).

#### Remarks.

The new species is easily distinguished from other species of the genus by the polymorphic abdominal tergites of the female. The fronto-ventral process of hypandrium is short and pointed. The biology of the species is unknown, but the female is saprophagous and the male is phototactic.

### Sinogodavaria tenebrosa sp. nov.

Taxon classificationAnimaliaDipteraPhoridae

CF67B9F9-03F6-5639-8C2A-72CF251E9976

http://zoobank.org/D1B0FA57-8ACF-418E-B7A2-E4DD741028B4

Figures 14–20 , 21–26

#### Specimens examined.

***Holotype***: China • ♀; Shaanxi, Zuoshui, Mt. Huanghua; 33°46'53.04″N, 108°49'37.91″E; alt. 1935 m; 14 Jul. 2013; Yunlong Cai leg.; sweeping net. ***Paratypes***: China • 1♀; Shaanxi, Zuoshui, Dagangou; 33°47'36.47″N, 108°55'38.17″E; alt. 1299 m; 15 Jul. 2013; Yunlong Cai leg.; sweeping net • 1♀1♂; Shaanxi, Zuoshui, Xigou; 33°49'27.98″N, 108°57'58.85″E; alt. 1197 m; 16 Jul. 2013; Yunlong Cai; sweeping net.

#### Diagnosis.

Female abdominal tergites uniformly dark brown; tergites II–III rectangular, tergites IV–VI trapezoid. Male tergites mostly dark brown, tergite VI with triangular yellow area at rear edge; hypandrium with a long hockey-stick-shaped fronto-ventral process.

**Figures 14–20.**
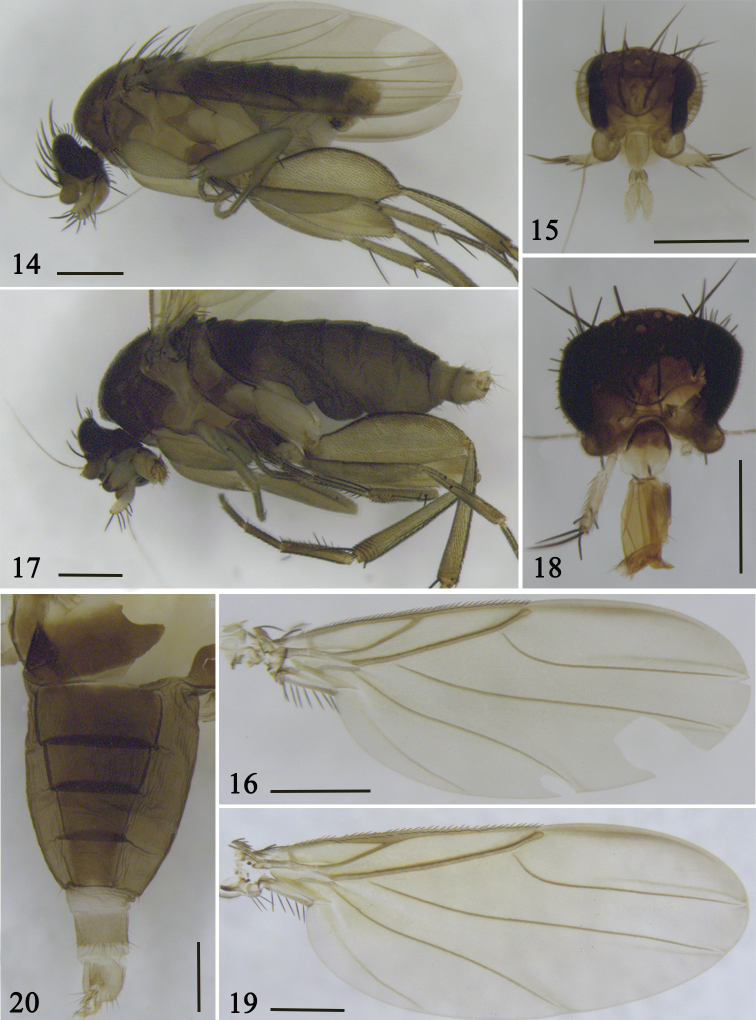
*S. tenebrosa* sp. nov. **14–16** Male **14** lateral view of body **15** head **16** wing **17–20** female **17** lateral view of body **18** head **19** wing **20** abdominal tergites. Scale bars: 0.5 mm.

#### Description.

**Female** (Fig. 17). ***Frons*** (Fig. 18) dark brown, a little broader than long, with 130–150 setulae and dense microsetae. Median furrow very short, vestigial. Lower interfrontal setae near to front edge, clearly close to each other and ca. 1/2 × as far apart as either is from a lower fronto-orbital seta, which is much higher on frons. Upper interfrontal setae as far apart as either is from an upper fronto-orbital seta, which is slightly higher on frons. Cheek with a single seta and jowl with two longer setae and some setulae. Flagellomere 1 globose, dark brown, with ca. 12 subcuticular pit sensilla; arista sub-apical, with microsetae; scape with dense microsetae. Palpus yellow, with six or seven apical setae and a dozen ventral setulae. Proboscis a little elongate. Labrum yellow, rectangular and a little wider than diameter of flagellomere 1. Labella straw yellow, each with ca. six setae on upper face and a submarginal row of ca. six small setulae below, and with five tracheae.

***Thorax*** mainly dark brown, being brown on sides. Anepisternum with fine setulae on upper part. Notopleura with four setae, the second being smaller than the rest. Scutellum with an anterior pair of small setulae and a posterior pair of setae.

***Legs*** yellow. Fore tibia with a near-dorsal seta at basal two fifths and five small setulae below it. Fore tarsus with a postero-dorsal longitudinal setal palisade on tarsomeres 1–3 only, plus a vestigial palisade in basal third of tarsomere 4. Mid tibia with the normal basal pair of setae and an antero-apical seta. Hind tibia with two dorsal longitudinal setal palisades, one antero-apical seta, one robust ventral spur, and a series of weak posterior and postero-dorsal spurs.

***Wing*** (Fig. 19) 3.28 mm long. Costal index 0.58. Costal ratio1:1.68. Costal cilia 45–50 µm long. Vein Rs with 18–20 pale and minute setulae along upper side. Costa thickened around the junction with vein R_1_. Vein Sc reaching vein R_1._ Axillary ridge with five long, black, feathered setae. Wing veins yellowish brown and membrane tinged yellowish grey. Haltere yellowish brown, knob black.

***Abdominal tergites*** (Fig. 20) uniformly dark brown, venter grayish brown. Tergites I–VI with very sparse short setulae and only a little longer at rear of tergite VI. Tergites II–III rectangular, tergites IV–VI trapezoid. Tergite II is the broadest and narrows gradually posteriorly. Venter greyish brown, with sparse setulae on segments V–VI. Cerci yellowish brown, ca. 3 × as long as broad.

**Male**(Fig. 14). ***Frons*** (Fig. 15) dark brown, a little broader than long, with 150–160 setulae and dense microsetae. Median furrow shorter than in female. Lower interfrontal setae near to front edge, clearly close to each other and ca. 2/3 × as far apart as either is from a lower fronto-orbital seta, which is much higher on frons. Upper interfrontal setae as far apart as either is from an upper fronto-orbital seta, which is slightly higher on frons. Proboscis shorter than in female. Labrum yellow with a very narrow triangle. Labella straw yellow, each with ca. eight setulae on upper face and a submarginal row of ca. 12 small setulae below.

***Thorax*** similar to female in color and chaetotaxy. Legs yellow. Front tibia with a near-dorsal seta at basal two-fifths and eight or nine small setulae below it. Front tip of hind coxa with a strong, feathered seta, which is more robust than in female. Wing (Fig. 16) 2.32 mm long. Costal index 0.53. Costal ratio 1:1.05. Costal cilia 45 µm long. Vein Rs with 16–18 pale minute setulae along upper side. Costa not thickened. Vein Sc reaching vein R_1_. Axillary ridge with five long, black, feathered setae. Wing veins yellowish brown and membrane tinged yellowish grey. Haltere yellowish brown, knob black.

***Abdominal tergites*** uniformly dark brown, but tergite VI with triangular yellow area at rear edge. Venter greyish brown, with several setulae on segments IV and V; setulae much longer than those on tergites. Tergites with very sparse short setulae, only a little longer at rear of tergite VI. Tergites II–VI rectangular or trapezoid. Tergite II broadest, others narrowing gradually. Cerci yellowish brown, ca. 3 × as long as broad.

**Figures 21–26.**
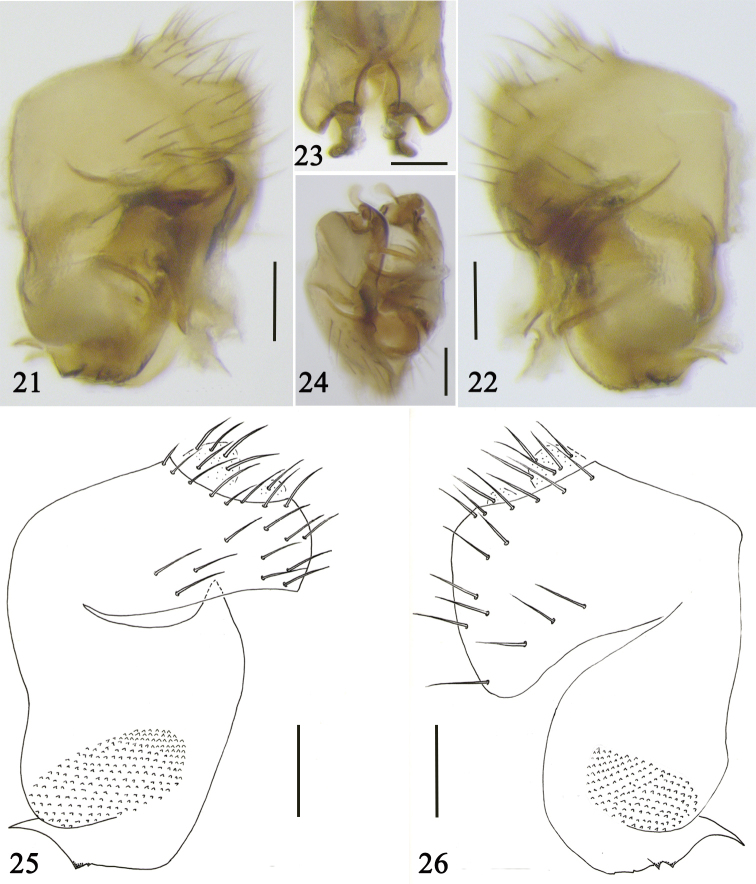
Male terminalia of *S. tenebrosa* sp. nov. **21–25** left view **22–26** right view **23** front view **24** ventral view. Scale bars: 0.1 mm.

***Terminalia*** (Figs 21–26). Left side of epandrium slender, rounded apically, with 20–25 setulae and dense microsetae; right side of epandrium large, triangular, with some setulae and dense microsetae. Left side of hypandrium short and wide, with a pointed upper corner. Right side of hypandrium short and rounded. A long hockey-stick-shaped process present at fronto-venter of each side of hypandrium. Aedeagus dark brown, supported by aedeagal apodeme and with a long, curled process. Cerci pale brown, short and with long setulae.

#### Etymology.

The name refers to the dark color of the species.

#### Distribution.

China (Shaanxi).

#### Remarks.

The new species is distinguished from *S. multiformis* by the uniform color, the shape of the female abdominal tergites, and the long hockey-stick-shaped process at the fronto-venter of the hypandrium.

### Sinogodavaria bathmis

Taxon classificationAnimaliaDipteraPhoridae

(Liu)

212312DE-D56A-5B43-A168-43A394F18755

Figures 27–29

Latiborophaga bathmis Liu, 2001: 39.

#### Specimen examined.

China • 1 ♀ (holotype); Sichuan, Baoxing, Mt. Huashu; 30°23'11.56″N, 102°49'59.16″E; alt. 1330 m; 2 Aug. 1992; Min Wang leg.; sweeping net.

#### Diagnosis.

Female abdominal tergites and venter uniformly black; tergite V rectangular, 2 × as long as its width; tergite VI triangular.

**Figures 27–29.**
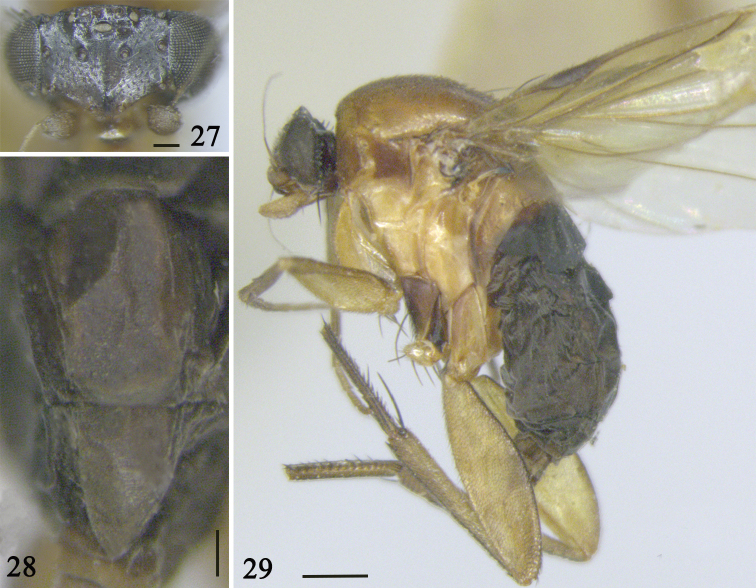
*S. bathmis* (Liu) **27** head **28** abdominal tergites V–VI **29** lateral view of body. Scale bars: 0.1 mm (**27–28**); 0.5 mm (**29**)

#### Description.

**Female** (Fig. 29). ***Frons*** (Fig. 27) fully black, broader than high, with 150–160 setulae and dense microsetae. Median furrow very short, vestigial. More than ten small setulae at frontal edge. Lower interfrontal setae near to front edge and closer to each other than either is to a lower fronto-orbital seta, which is much higher on frons. Upper interfrontal setae as far apart as either is from the upper fronto-orbital seta, which is higher on frons. Flagellomere 1 dark brown, globose; arista sub-apical, covered with minute setulae. Raised lower margin of face. Palpus 0.36 mm long, light brown, with seven apical setae and several ventral setulae. Proboscis elongate. Labrum yellow, rectangular, a little wider than diameter of flagellomere 1. Labella straw yellow, each with ca. six setulae on upper face and a submarginal row of ca. 12 small setulae below, with five tracheae.

***Thorax*** dark brown, being paler on sides. Anepisternum with fine setulae on upper part. Notopleura with four setae, the second being smaller than the rest. Scutellum with an anterior pair of small setulae and a posterior pair of setae.

***Legs*** yellow, fore tibia with a near-dorsal seta at basal two fifths and five differentiated small setulae below it. Fore tarsus with a postero-dorsal longitudinal setal palisade on tarsomeres 1–3 only, plus a vestigial palisade in basal one third of tarsomere 4. Mid tibia with the normal basal pair of setae and an antero-apical seta. Hind tibia with two dorsal longitudinal setal palisades, of which the posterior one extends to four fifths of tibia, and with one antero-apical seta, one robust ventral spur, and a series of weak posterior and postero-dorsal spurs.

***Wing*** length 3.75 mm, slightly greyish yellow, veins brown. Costal index 0.6. Costal ratio 1: 2.04. Costal thickening black. Vein Rs with 20 pale minute setulae along upper side. Axillary ridge with four long, black, feathered setae. Wing veins yellowish brown and membrane tinged yellowish grey. Haltere yellow with black knob.

***Abdominal tergites*** and venter uniformly black. Tergite II broadest, the others narrowing gradually. Tergites III and IV trapezoid; tergite V rectangular, longer than broad; tergite VI triangular (Fig. 28). Tergites with very sparse setulae. Abdominal venter black.

**Male.** Unknown.

#### Distribution.

China (Sichuan).

#### Remarks.

The species differs from other known species of the genus by the rectangular shape of tergite V and the triangular tergite VI. It was formerly assigned to the genus *Latiborophaga*; however, close examination indicates that the species does not belong to the genus *Latiborophaga* due to the vein Rs not being deflected at mid length, hind tibia without an antero-basal seta, and hypandrium without dense microsetae.

## Discussion

Brown (1992) considered that the genus *Borophaga* Enderlein, 1924 in the sense of Schmitz (1927; 1929; 1951) was polyphyletic and split the genus into four genera: *Borophaga*, *Godavaria* Brown, 1992, *Latiborophaga* Brown, 1992, and *Peromitra* Enderlein, 1924. Liu (1996) erected the new genus *Chaetogodavaria*, which is closely related to *Godavaria*. In the phylogenetic analysis of Brown et al. (2015), the *Hypocera* group, including *Hypocera* Lioy, 1864, *Borophaga* Enderlein, 1924, *Abaristophora* Schmitz, 1927, *Latiborophaga* Brown, 1992, *Godavaria* Brown, 1992, *Chaetogodavaria* Liu, 1996, *Stichillus* Enderlein, 1924, *Peromitra* Enderlein, 1924 and *Trineurocephala* Schmitz, 1923, was proposed based on the character states of an extremely reduced lumen of the hind coxa and the rounded spinuli of the hypandrium. The new genus described in this work may belong to the *Hypocera* group; however, the details of its phylogeny are unknown, because no synapomorphic character was found. Compared to the phylogenetic analysis by Brown et al. (2015: fig. 47), the new genus can converge to *Godavaria* and *Chaetogodavaria* based on the character of an antero-apical seta on the hind tibia (feature 88). It can also converge to *Latiborophaga* based on the character of the postpronotal seta (feature 44).

## Supplementary Material

XML Treatment for Sinogodavaria

XML Treatment for Sinogodavaria multiformis

XML Treatment for Sinogodavaria tenebrosa

XML Treatment for Sinogodavaria bathmis
